# Sleep Deprivation Does not Change the Flash Electroretinogram in Wild-type and *Opn4^−/−^Gnat1^−/−^* Mice

**DOI:** 10.1177/07487304221074995

**Published:** 2022-02-08

**Authors:** Robin A. Schoonderwoerd, Thilo M. Buck, Charlotte A. Andriessen, Jan Wijnholds, Samer Hattar, Johanna H. Meijer, Tom Deboer

**Affiliations:** * Laboratory for Neurophysiology, Department of Cell and Chemical Biology, Leiden University Medical Center, Leiden, the Netherlands; † Department of Ophthalmology, Leiden University Medical Center, Leiden, the Netherlands; ‡ Section of Light and Circadian Rhythms, National Institutes of Health, Bethesda, Maryland, USA

**Keywords:** sleep, electroretinogram, photopic, scotopic, sleep deprivation, circadian clock

## Abstract

Sleep deprivation reduces the response of neuronal activity in the suprachiasmatic nucleus (SCN) and the phase shift in circadian behaviour to phase shifting light pulses, and thus seems to impair the adaptation of the circadian clock to the external light-dark cycle. The question remains where in the pathway of light input to the SCN the response is reduced. We therefore investigated whether the electroretinogram (ERG) changes after sleep deprivation in wild-type mice and in *Opn4^−/−^Gnat1^−/−^* mutant male mice. We found that the ERG is clearly affected by the *Opn4^−/−^Gnat1^−/−^* mutations, but that the ERG after sleep deprivation does not differ from the baseline response. The difference between wild-type and mutant is in accordance with the lack of functional rod and melanopsin in the retina of the mutant mice. We conclude that the decrease in light responsiveness of the SCN after sleep deprivation is probably not caused by changes at the retinal level, but rather at the postsynaptic site within the SCN, reflecting affected neurotransmitter signalling.

Light is the most important environmental cue to entrain the biological clock. Light is sensed in the retina by a subset of intrinsically photosensitive retinal ganglion cells (ipRGCs), which contain the photopigment melanopsin. In addition, ipRGCs incorporate input from rod and cone photoreceptors (Guler et al., 2008). The ipRGCs project via the retinohypothalamic tract (RHT) to the suprachiasmatic nucleus (SCN) of the hypothalamus, the location of the circadian clock in mammals, enabling photoentrainment of the SCN circadian pacemaker (Moore and Lenn, 1972). Following activation by light, glutamate is released at the nerve terminal (Johnson et al., 1988; Ding et al., 1994), which leads to an increase in SCN neuronal activity (Meijer et al., 1992, 1998; Cui and Dyball, 1996; Aggelopoulos and Meissl, 2000; Nakamura et al., 2004; Drouyer et al., 2007; Van Oosterhout et al., 2012; Van Diepen et al., 2013, 2014, 2021). The increase in electrical activity in the SCN in response to light consists of a transient onset response and a subsequent sustained response, where activity remains increased for the total duration of the light pulse (Meijer et al., 1998).

Light exposure at the beginning of the night delays the circadian rest-activity rhythm. Sleep deprivation attenuates both this phase shifting effect on behavioural rest-activity (Mistlberger et al., 1997; Challet et al., 2001; Van Diepen et al., 2014) and the increase in SCN neuronal activity (Van Diepen et al., 2014) in response to light. The attenuated SCN neuronal activity during light exposure seemed to mainly consist of a reduction in the sustained response to light as light onset and offset still were able to induce transient responses (Van Diepen et al., 2014, Figure 2). Sleep deprivation therefore seems to impair the ability of the central circadian pacemaker to adapt to the external light-dark cycle. The first explanation for this was that sleep deprivation affects central processing of light information (Van Diepen et al., 2014). This would be in line with the reduction in SCN neuronal activity following sleep deprivation (Deboer et al., 2007). However, the possibility that sleep deprivation affects signalling at the retinal level could not be excluded.

There is a large resemblance in the reduction in the phase shift in rest-activity behaviour and the sustained response in electrical activity in the SCN to the changes seen in these parameters in *Opn4^−/−^Gnat1^−/−^* mice. These mice lack functional rod signalling, due to a targeted deletion of the rod transducing alpha subunit *Gnat1*, and melanopsin in the ipRGCs, due to the *Opn4* knockout (Mrosovsky and Hattar, 2005). As a consequence, the mutant mice show a reduction in phase shifting capacity and the sustained electrophysiological response in the SCN to the visible light wavelengths (Van Diepen et al., 2021).

To investigate whether the reduction in circadian light responses in animals under increased sleep pressure originates from similar changes in retinal function, as induced in *Opn4^−/−^Gnat1^−/−^* mice, we set out to record the electroretinogram (ERG) in wild-type mice and mutant mice, both under baseline conditions and after a sleep deprivation. If the effect of sleep deprivation on SCN neuronal responses and phase shifting capacity is caused by changes at the outer retinal level, the ERG after sleep deprivation in wild-type mice is expected to become more similar to the ERG in *Opn4^−/−^Gnat1^−/−^* mice. In addition, the ERG in the mutant mice should not be affected by sleep deprivation. We show that the ERG in *Opn4^−/−^Gnat1^−/−^* mice differs from wild-type mice, but that the ERG does not change in either of the two groups of mice after sleep deprivation. Therefore, sleep deprivation-induced changes in circadian functioning seen in previous research are probably not caused by changes in the outer retinal response to light.

## Materials and Methods

### Animals

The experiments were approved by the Central Committee Animal Research. All experiments were carried out in accordance with EU Directive 2010/63EU on the protection of animals used for scientific purposes. *Opn4^−/−^Gnat1^−/−^* mice originated from the lab of Prof Samer Hattar (Johns Hopkins University, Baltimore) and were backcrossed on a C57/Bl6JIco background at the Leiden University Medical Center. Experiments were carried out with male (*n* = 5, age: 2-5 months) with homozygous knockouts of the *Opn4* and *Gnat1* genes. Wild-type animals (*n* = 5 age: 2-5 months) from the same background were obtained from Charles River to minimize differences, apart from potential developmental alterations.

### ERG Recordings

Dark- and light-adapted ERGs were performed under dim red light using an Espion E2 (Diagnosys, LLC, Lowell MA, USA). Mice were anesthetized using 100-mg/kg ketamine and 10-mg/kg xylazine administered intraperitoneally, and the pupils were dilated using tropicamide drops (5 mg/mL). Mice were placed on a temperature regulated heating pad, and reference and ground platinum electrodes were placed subcutaneously in the scalp and the base of the tail, respectively. ERGs were recorded from both eyes using gold wire electrodes. Hypromellose eye drops (3 mg/mL, Teva, the Netherlands) were given between recordings to prevent eyes from drying. Single (scotopic and photopic ERG) white (6500 k)-flashes were used with an interstimulus interval of 5 s. Band-pass filter frequencies were 0.3 and 300 Hz. Scotopic recordings were obtained from dark-adapted animals at the following light intensities: −4, −3, −2, −1, 0, 1, 1.5, and 1.9 log cd·s/m^2^. Photopic recordings were performed following 10-min light adaptation on a background light intensity of 30 cd·m^2^ and the light intensity series used was −2, −1, 0, 1, 1.5, and 1.9 log cd·s/m^2^ (Nishiguchi et al., 2015; Quinn et al., 2019). Responses of the *Opn4^−/−^Gnat1^−/−^* mice were compared to the wild-type control mice at each light condition analysed.

### Sleep Deprivation

Sleep deprivation was performed in groups of 5 mice in constant dim red light to keep the animals dark adapted. During the sleep deprivation, the animals were kept awake by the researcher. Every time the mice appeared drowsy, they were mildly disturbed by noise, movement of bedding, or the introduction of new nesting material (Deboer et al., 2013; Van Diepen et al., 2014; Panagiotou et al., 2017). ERGs were performed after the animals had been awake for at least 6 h. The animals were kept awake until the time of recording. The data were compared to ERGs recorded during an undisturbed baseline day obtained at the same time of day in the same animals within the same week.

### Statistics

All statistical analyses were performed using GraphPad Prism version 7 (GraphPad software). We performed a two-way analysis of variance (ANOVA) with a Bonferroni post hoc test (after averaging right and left eye of a mouse). All values are expressed as mean ± standard error of the mean (SEM) if not otherwise indicated. Statistical significant values are as follows: *p < 0.05, **p < 0.01, ***p < 0.001.

## Results

**Figure 1.**
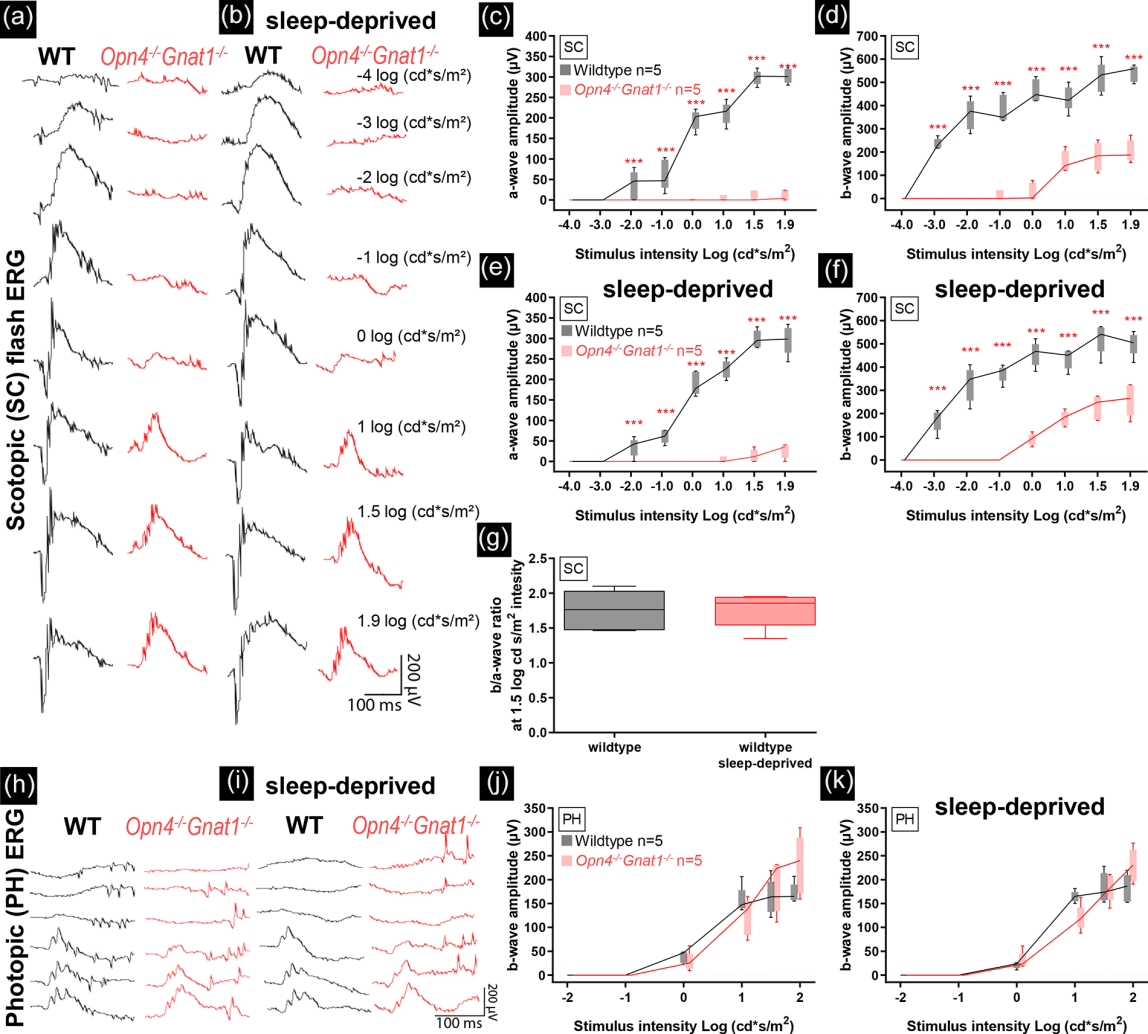
The *Opn4^−/−^Gnat1^−/−^* knockout caused severe loss of retinal scotopic functioning (dark-adapted. Flash-intensity series: −4, −3, −2, −1, 0, 1, 1.5, and 1.9 log cd·s/m^2^), but left photopic (light-adapted. Flash-intensity series: −2, −1, 0, 1, 1.5, and 1.9 log cd·s/m^2^) functioning intact. Sleep deprivation did not change retinal functioning in wild-type control and *Opn4^−/−^Gnat1^−/−^* mice. Scotopic (a, b) and photopic (h, i) electroretinographic analysis of a representative control (black) and *Opn4^−/−^Gnat1^−/−^* mouse (red) before sleep deprivation. Reduction in scotopic single-flash intensity a-wave (c, e) and b-wave amplitudes (d, f) plotted as a function of the logarithm of the flash intensity were found in *Opn4^−/−^Gnat1^−/−^* mice. No differences were found in photopic a- and b-wave amplitudes (j, k). No effect of sleep deprivation was found (e, f, g, k). Boxes indicate the 25% and 75% quantile range and whiskers indicate the 5% and 95% quantiles, and the intersection of line and error bar indicates the median of the data (box-and-whisker plot). Number of animals used: 5 controls and 5 *Opn4^−/−^Gnat1^−/−^* mice. Abbreviation: ERG = electroretinogram: WT= wild-type. *p < 0.05. **p < 0.01. ***p < 0.001.

We analysed *Opn4^−/−^Gnat1* mice and age-matched control animals by ERG (representative traces shown in Figure 1a and 1h). The *Opn4^−/−^Gnat1^−/−^* mice showed a significant loss of retinal activity compared to age-matched control animals in the scotopic a-wave—two-way ANOVA; *F*(1,64) = 1101; *P* < 0.001; Figure 1c—and b-wave—*F*(1,64) = 969.5; *P* < 0.001; Figure 1d—whereas the photopic response was not affected, *F*(1,48) = 0.8212; *P* = 0.369; Figure 1j, confirming alterations in the *Opn4^−/−^Gnat1^−/−^* mice in the rod system components. At high stimulus intensities under scotopic conditions, the a-wave of the *Opn4^−/−^Gnat1^−/−^* animals seemed comparatively more reduced than the b-wave. Our results indicate that the retinas of *Opn4^−/−^Gnat1^−/−^* mice have a severe functional impairment in the rod system, whereas cone responses are normal.

The measurements were repeated at the end of a 6-h sleep deprivation in both wild-type and *Opn4^−/−^Gnat1^−/−^* mice (representative traces shown in Figure 1b and 1i). Sleep deprivation did not change the scotopic or photopic response in either of the two mouse genotypes. We therefore conclude that sleep deprivation does not change outer retinal functioning in ERG measurements.

## Discussion

The retinal activity of the *Opn4^−/−^Gnat1^−/−^* mice was substantially decreased for the a-wave (rod response), as well as the b-wave (summed rod and inner retinal response) in the scotopic flash stimuli, whereas the photopic (cones) response was not affected. The ERG data obtained in these mice are in agreement with a complete loss of rod signalling, but with intact cone mechanisms. Following the sleep deprivation, no changes were observed in the ERG of *Opn4^−/−^Gnat1^−/−^* mice or in wild-type control, confirming that the influence of sleep deprivation on SCN light responses is due to changes in central processing of light information.

### Opn4^−/−^Gnat1^−/−^ and the Reduction in Phase Shifting Capacity

*Opn4^−/−^Gnat1^−/−^* mice were shown to have reduced phase shifting capacity and a reduced sustained response in SCN neuronal activity when exposed to a phase shifting light pulse (Van Diepen et al., 2021). The previous data suggested that mouse cone subtypes (S-cone vs M-cone) have distinct contributions to photoentrainment and SCN electrical activity, with the medium (green light) wavelength sensitive (M)-cone having less influence compared to the UV short wavelength sensitive (S)-cone. In this study, we investigated how the mutations may influence the signalling from the eye to the brain by recording ERG under scotopic and photopic white light pulses. Our data confirm preliminary studies published previously (Lall et al., 2010) and show that in the scotopic range, the response in the *Opn4^−/−^Gnat1^−/−^* mice is absent or greatly reduced, which is in accordance with the lack of functional rod signalling in these mice. Studies have reported minimal residual rod activation at very high light intensities (Calvert et al., 2000) through the Gnat2 signalling pathway (Allen et al., 2010). In our data, this effect is also shown by the slight increase of the scotopic a-wave and the presence of the b-wave at light intensities higher than 1.0 cd·s/m^2^. The photopic response was not affected by the mutations, which can be expected as this response is mainly mediated by the cones, which are intact and functioning in the mutant mice. The data show that the cone response to light is intact in these animals, whereas the rod response is virtually absent.

### Sleep Deprivation and the Reduction in Phase Shifting Capacity

In the second part of the experiment, we sleep deprived the mice to investigate whether sleep deprivation reduces the retinal activity to light stimuli (ERG flashes) in wild-type mice. In previous studies, it was shown that sleep deprivation attenuates the phase shift in rest-activity behaviour in response to light and the electrophysiological response in the SCN (Mistlberger et al., 1997, Challet et al., 2001; Van Diepen et al., 2014). Treatment with caffeine was able to restore the SCN electrophysiological response (Van Diepen et al., 2014). Behavioural studies showed that caffeine likely increases SCN susceptibility to light as the phase shifting capacity to a light pulse increased (Ruby et al., 2018; Jha et al., 2017), and circadian period lengthened under constant light conditions (Van Diepen et al., 2014). The effects of caffeine support the suggestion that adenosine, which accumulates in extracellular space due to increased neuronal activity, particularly due to prolonged waking (Porkka-Heiskanen et al., 1997; Landolt, 2008), attenuates the light input to the SCN. This most likely is by reducing glutamate release at the transition from the RHT to the SCN (Van Diepen et al., 2014), but might also be due to a reduced response to light in the retina. Here we show that the ERG did not change when performed at the end of a 6-h sleep deprivation. This means that there are no substantial changes in the rod or cone flash responses after sleep deprivation, but alterations in retinal responses to longer pulses of light are not detectable in the flash ERG and cannot be excluded. Nevertheless, the present results reduce the likelihood that changes in retinal activity in response to light are the cause of the attenuated phase shifting capacity in rest-activity behaviour and reduced electrophysiological responses to light in the SCN after sleep deprivation.

In conclusion, we found that the neurobiological mechanisms underlying reduced circadian phase shifting and diminished sustained neuronal activation in the SCN differ between *Opn4^−/−^Gnat1^−/−^* mice and sleep deprived wild-type animals. In *Opn4^−/−^Gnat1^−/−^* mice, the alterations have a retinal origin as expected, whereas in sleep deprived mice, the changes likely occur downstream from the eyes. Since the effect of sleep deprivation is visible in the electrophysiological light response of SCN neuronal activity (Van Diepen et al., 2014), the effect of sleep deprivation on the phase shifting capacity of the central circadian pacemaker to light is therefore due to changes in maintained activity in the retina, or a reduced postsynaptic responsiveness of the SCN to light.
